# Spatially varying gene regulation network inference from spatial transcriptomics

**DOI:** 10.1093/bioadv/vbag230

**Published:** 2026-08-14

**Authors:** Yurui Li, Jin Chen, Ting Lu, Nien-Pei Tsai, Haohan Wang

**Affiliations:** School of Information Sciences, University of Illinois Urbana-Champaign, Urbana, IL 61820, United States; Department of Inflammation and Immunity, Lerner Research Institute, Cleveland Clinic, Cleveland, OH 44195, United States; Department of Bioengineering, The Grainger College of Engineering, University of Illinois Urbana-Champaign, Urbana, IL 61820, United States; Department of Molecular and Integrative Physiology, School of Molecular & Cellular Biology, College of Liberal Arts & Sciences, University of Illinois Urbana-Champaign, Urbana, IL 61820, United States; School of Information Sciences, University of Illinois Urbana-Champaign, Urbana, IL 61820, United States

## Abstract

**Motivation:**

Gene regulatory networks (GRNs) govern cellular functions by coordinating gene expression programs. These regulatory relationships are strongly shaped by local microenvironments, giving rise to dynamic, spatially varying regulatory patterns across tissues. Therefore, it is crucial to infer GRNs at higher, cell-specific resolution while jointly modeling spatial context. However, most existing GRN inference approaches focus on cell-type–level networks or infer cell-specific GRNs without incorporating neighborhood and positional information.

**Results:**

We propose SVGRN, a deep learning framework for inferring spatially resolved, high-resolution GRNs from spatial transcriptomics data. SVGRN integrates gene expression, regulatory interactions, and spatial coordinates within a structural equation modeling framework implemented by a conditional variational autoencoder, to learn nonlinear, spatially varying regulatory programs in an unsupervised manner. By conditioning on target locations and incorporating neighborhood information, SVGRN refines tissue-level regulation into spot- or cell-specific GRNs. Across simulated datasets, SVGRN consistently outperforms existing methods under diverse and challenging settings. Applications to seqFISH mouse embryo data and Visium human cutaneous squamous cell carcinoma and fallopian tube datasets demonstrate that SVGRN captures spatially varying regulatory programs underlying development, tumor progression, and tissue organization, highlighting its robustness and broad applicability.

**Availability and implementation:**

The source code and data are available at https://github.com/lyrrrr/SVGRN.

## 1 Introduction

The collaboration between chromatin, transcription factors, and genes forms complex regulatory circuits that can be modeled as gene regulatory networks (GRNs) (Badia-I Mompel *et al.* 2023). Unraveling these interactions is critical for understanding the regulatory crosstalk underlying cellular processes and diseases (Kim *et al.* 2023). Rather than being static within tissues, GRNs vary across cells in response to distinct microenvironmental inputs, including spatial positioning, neighboring cell composition, and intercellular signaling, which collectively shape heterogeneous regulatory programs (Movasat *et al.* 2025, Pareja-Lorente and Aloy 2025). Therefore, studying GRN heterogeneity requires accounting for spatial context. Spatial transcriptomics enables this by jointly measuring gene expression and cellular locations, allowing integrative analysis of each cell’s microenvironment and its own gene activities.

With the rapid development of transcriptomics technologies, numerous computational methods have been proposed to infer GRNs at progressively higher resolution. Early approaches based on bulk transcriptomic data (Margolin *et al.* 2006, Langfelder and Horvath 2008) obscure more specific regulatory programs by aggregating heterogeneous cell populations. The emergence of single-cell and spatial transcriptomics has enabled GRN inference across cell types and differentiation trajectories. Many single-cell-based methods (Huynh-Thu *et al.* 2010, Chan *et al.* 2017, Moerman *et al.* 2019, Shu *et al.* 2021) infer cell-type–level networks by aggregating expression across cells, limiting their ability to capture individual-cell-level regulatory heterogeneity. Only a few methods infer cell-specific networks, using gene coexpression (Dai *et al.* 2019, Li *et al.* 2021, Wang *et al.* 2021), causal inference (Chang *et al.* 2024a, Huang *et al.* 2025), or dynamic and information-theoretic frameworks (Zhang and Stumpf 2025). In parallel, spatial transcriptomics-based methods (Acharyya *et al.* 2022, Littman *et al.* 2023, Chang *et al.* 2024b, Li *et al.* 2025b), incorporate spatial information but primarily infer cell-type–level networks. CeSpGRN (Zhang *et al.* 2022) extends this by inferring spot- or cell-specific GRNs using Gaussian graphical models with kernel-based smoothing.

However, existing methods remain limited in capturing how GRNs are shaped by complex spatial microenvironments. In tissues, GRNs vary across space and are strongly influenced by local neighborhood context, making cell-level GRN inference within spatial settings essential. Current approaches either focus on cell-type-level networks, overlooking context-dependent regulatory variation among cells of the same type—such as differences between tumor-core and tumor-edge cells (Arora *et al.* 2023) or among cells located at different cortical depths (Wei *et al.* 2025)—that is important for understanding how local microenvironments shape tissue development and disease progression, or infer cell-specific GRNs mainly from latent gene expression similarities without fully incorporating spatial organization. In addition, many rely on linear or kernel-based formulations, restricting their ability to model nonlinear regulatory programs and complex microenvironmental effects.

Therefore, we propose SVGRN, a deep learning framework for inferring spatially varying, spot- or cell-specific GRNs from spatial transcriptomics data. By leveraging spatial coordinates and neighborhood information, SVGRN refines tissue-level regulatory patterns into individual unit-specific GRNs that reflect heterogeneous microenvironmental context. SVGRN employs a conditional variational autoencoder (CVAE) to model gene expression and cellular layout, enabling flexible nonlinear modeling of spatially varying regulatory programs. Across multiple simulated datasets, SVGRN outperforms representative baseline methods under diverse settings. Applications to cell-based mouse embryo data demonstrate its ability to uncover smoothly varying regulatory programs across tissue landscapes during early organogenesis. Furthermore, analyses of spot-based cSCC and fallopian tube datasets reveal biologically consistent GRNs that reflect microenvironment-driven regulatory heterogeneity, highlighting the robustness and broad applicability of SVGRN across platforms.

## 2 Methods

### 2.1 Gene interaction modeling with spatial coordinates

To unify single-cell and spot-based spatial transcriptomics settings, we define a spatial unit as a cell or spot here. SVGRN infers a GRN matrix for each spatial unit given its gene expression and spatial coordinates. Biologically, gene expression arises from regulatory interactions, where each gene can be modeled as a function of its regulators and noise. This motivates a structural equation modeling (SEM) framework, which represents each variable as a function of others. In the GRN inference problem setting, let $X\in R^{n\times m}$ be the gene expression matrix with *n* cells and *m* genes and $W\in R^{m\times m}$ be the GRN adjacency matrix that represents the dependencies among different genes. The relationship between X and W is formalized following the basic linear SEM as


(1) $$X=XW^{T}+Z$$

where $Z\in R^{n\times m}$ is a Gaussian noise matrix capturing transcriptional variability and measurement noise. This formulation models each gene’s expression as a linear combination of other genes. The (1) can be modified to


(2) $$X=Z{\left(I-W^{T}\right)}^{-1}$$

(3) $$Z=X(I-W^{T})$$

While linear SEM captures basic gene dependencies, gene regulation in biological systems is often more complex. In transcriptomics data, interactions involve diverse combinatorial and context-dependent factors, such as external environmental responses and cell–cell signaling, which are difficult to model with linear formulations. Therefore, we extend (2) and (3) to a nonlinear SEM (Yu *et al.* 2019) to better capture this complexity:


(4) $$X=f_{1}(Z{\left(I-W^{T}\right)}^{-1})$$

(5) $$Z=f_{2}(X)(I-W^{T})$$

where $f_{1}$ and $f_{2}$ are parameterized functions performing nonlinear transforms on $Z{\left(I-W^{T}\right)}^{-1}$ and X, respectively.

In spatially organized environments, cellular behavior is influenced by positional context. A cell’s location affects gene activation through signals from neighboring cells, shaping its development and function (Perrimon *et al.* 2012). During embryonic development, positions within morphogen concentration gradients further induce distinct regulatory programs (Kerszberg and Wolpert 2007). Spatial transcriptomics jointly measures gene expression and spatial location, enabling incorporation of positional information into GRN modeling. By integrating spatial coordinates, the model captures how location influences gene expression and reflects microenvironmental effects on cell function and differentiation. Accordingly, based on (4) and (5), we introduce $Y\in R^{n\times 2}$ to represent spatial coordinates, leading to the following formulation:


(6) $$X=f_{1,1}\left(f_{1,2}(Z,Y){\left(I-W^{T}\right)}^{-1}\right)$$

(7) $$Z=f_{2,2}\left(f_{2,1}(X)(I-W^{T}),Y\right)$$

where $f_{1,1}$, $f_{1,2}$, $f_{2,1}$, and $f_{2,2}$ are nonlinear functions parameterized by multilayer neural networks. Incorporating Y into $f_{1,2}$ and $f_{2,2}$ allows the model to capture nonlinear dependencies between gene expression and spatial organization while preserving gene regulatory relationships.

Equations (4)–(7) extend the linear SEM into nonlinear forward and reverse mappings while preserving the underlying gene regulatory structure. Equations (4) and (5) transform raw gene expression into nonlinear feature representations before GRN modeling to represent latent biological states beyond transcript abundance alone, whereas (6) and (7) incorporate spatial coordinates as additional information outside the GRN transformation to capture location-dependent, microenvironment-associated variation while preserving the role of the GRN layer. Together, these formulations provide a unified framework that jointly models nonlinear transcriptional regulation, spatial context, and gene regulatory networks, which aligns with the symmetry encoder–decoder architecture of the CVAE architecture described next.

### 2.2 SVGRN framework based on CVAE

To integrate (6) and (7), we adopt a CVAE (Sohn *et al.* 2015) to model the relationships among gene expression, GRNs, and spatial coordinates. The CVAE consists of an encoder that maps inputs to latent representations and a decoder that reconstructs data conditioned on additional information, corresponding to (7) and (6), and implemented with neural networks to capture nonlinear dependencies. Spatial coordinates Y are incorporated as conditional information, serving as an observable proxy for the cellular microenvironment under the biological assumption that nearby cells are more likely to share similar local signaling, neighboring cell compositions, and morphogen gradients. This enables the model to learn the correspondence between spatial context and transcriptional states. Given gene expression X and spatial coordinates Y, the encoder learns the latent distribution $q_{\phi }(Z|X,Y)$, parameterized by ϕ, capturing joint transcriptional and spatial information. The decoder $p_{\theta }(X|Z,Y)$, parameterized by θ, reconstructs gene expression conditioned on latent regulatory structure and spatial location. The GRN matrix W is jointly optimized as shared learnable parameters in the encoder and decoder, with its values modulated through spatially varying conditioning, and the converged W serves as the inferred GRN. By applying W to nonlinear gene representations within the spatially conditioned CVAE, the overall framework can capture nonlinear and context-dependent regulatory relationships, while the structured matrix multiplication retains a clear association between the learned weights and gene regulatory interactions.

**Figure 1:**
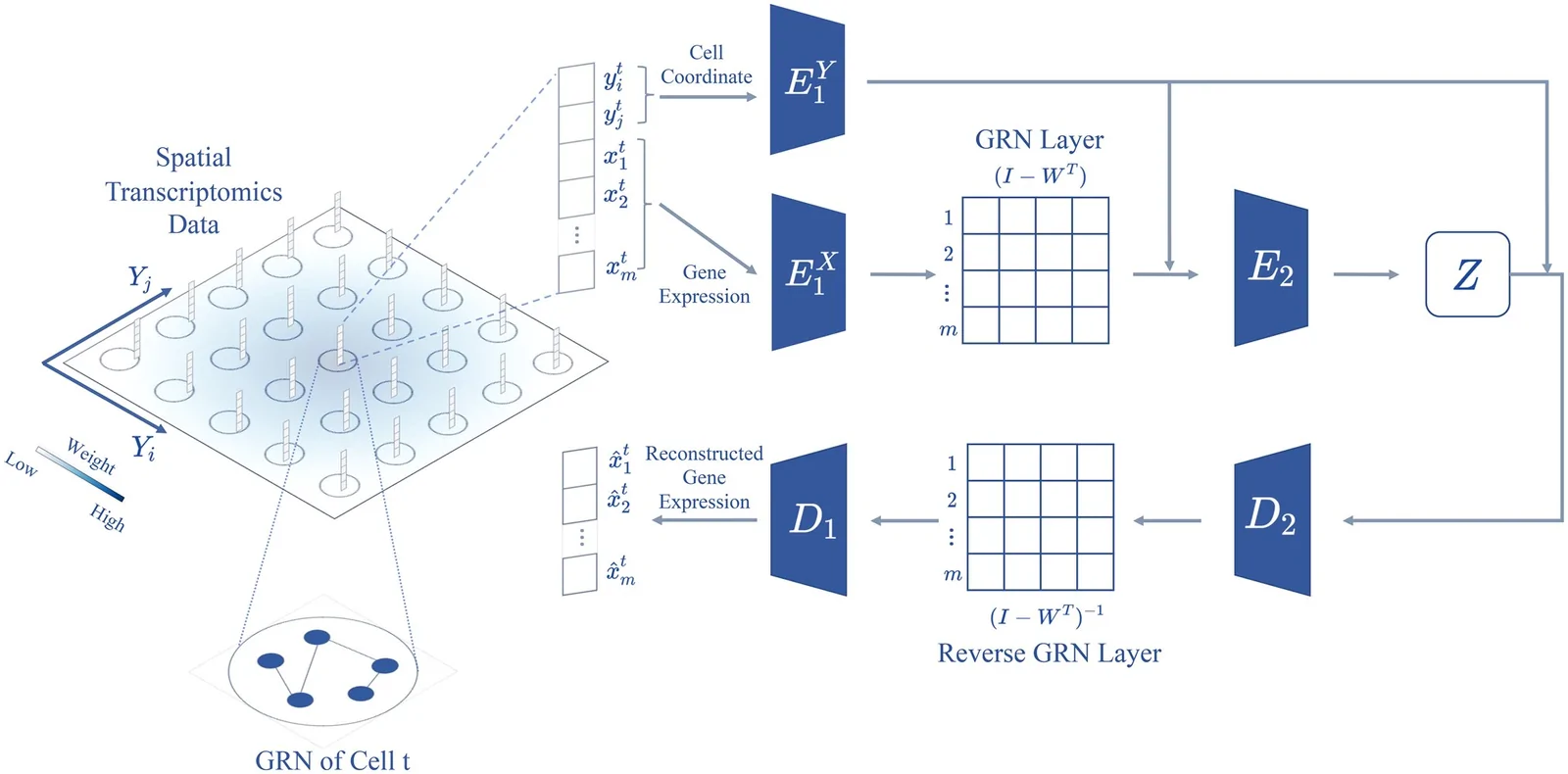
The SVGRN model architecture. The model takes the gene expression profile and spatial coordinates of each spatial unit as input. Gene expression is processed by $E_{1}^{X}$ and the GRN layer, while spatial coordinates are encoded by $E_{1}^{Y}$, with both representations integrated by $E_{2}$ into the latent space. For reconstruction, the latent representation is concatenated with the spatial embedding and passed sequentially through $D_{2}$, the inverse GRN layer, and $D_{1}$, with both GRN layers sharing the learnable matrix W. The model is trained in two stages: Stage 1 jointly optimizes all components to learn a global GRN, whereas Stage 2 freezes the encoders and decoders and optimizes only the GRN and inverse GRN layers for each target spatial unit to obtain unit-specific GRNs.

The SVGRN architecture (Fig. 1) follows our SEM formulation. The gene expression encoder $E_{1}^{X}$, corresponding to $f_{2,1}(\cdot )$ in (7), encodes expression features, while the coordinate encoder $E_{1}^{Y}$ maps the raw spatial coordinates Y into spatial feature representations. This implements the spatial conditioning term in (6)–(7) by introducing an additional coordinate encoder $E_{1}^{Y}$, which enables feature-level integration between spatial and gene-expression information. The output of $E_{1}^{X}$ passes through a GRN layer to model gene–gene dependencies. The network $E_{2}$, implementing $f_{2,2}(\cdot )$, integrates expression and coordinate features into the latent space. Symmetrically, $f_{1,1}(\cdot )$ and $f_{1,2}(\cdot )$ are implemented as decoders $D_{1}$ and $D_{2}$. $D_{2}$ decodes latent and coordinate features and applies a reverse GRN layer to remove gene dependencies, followed by $D_{1}$ to reconstruct gene expression. To ensure gene–gene interactions are fully captured within GRN layers, all other network components operate gene-wise with shared weights and no cross-gene mixing.

Given a dataset $D={\left\{(x^{k},y^{k})\right\}}_{k=1}^{n}$, where $x^{k}=X_{k,:}$ and $y^{k}=Y_{k,:}$ denote the gene expression and spatial coordinates of the *k*-th unit, the CVAE models the conditional distribution p(x∣y). This enables recovery of unit-specific gene expression conditioned on spatial location, thereby linking transcriptional patterns to cellular positions. SVGRN further extends the CVAE loss with an $L_{1}$ regularization term on the GRN adjacency matrix W:


(8) $$\begin{array}{cc} L=-E_{q_{\phi }(Z|X,Y)}[\text{log}\;p_{\theta }(X|Z,Y)] & \\ +\beta KL(q_{\phi }(Z|X,Y)\|p(Z|Y))+\alpha \|W{\|}_{1} & \end{array}$$

where *E* denotes the expectation, KL(·|·) is the Kullback–Leibler divergence, and α and β are hyperparameters. The $L_{1}$ regularization promotes sparsity in the learned GRN matrix W, reflecting the biological assumption that only a subset of genes directly regulate one another.

### 2.3 SVGRN training for spatial-unit-specific GRNs

SVGRN is trained in two stages that gradually refine regulatory inference from tissue-level to spot/cell-specific. Stage 1 learns a global GRN from all units using gene expression and spatial coordinates, capturing overall tissue-wide patterns. Stage 2 refines this network separately for each target unit by optimizing the model under its corresponding spatial location and spatially weighted neighboring information, resulting in a unit-specific GRN matrix for the target local microenvironment. This two-stage design establishes a biologically meaningful hierarchy for spatially resolved, unit-specific GRN inference. Details are provided below. The detailed pseudocode for the two-stage training procedure is summarized in Supplementary Note 3, available at supplementary material  *Bioinformatics Advances* online.

In the first training stage, the training dataset $D_{1}={\left\{(x^{k},y^{k})\right\}}_{k=1}^{n}$ is composed of pairs of gene expression and corresponding coordinates for each unit *k*. The loss function follows the same form of (8) and can be further written as follows


(9) $$\begin{array}{cc} L_{\mathrm{stage}1}=\frac{1}{n}\sum_{k=1}^{n}L(x^{k},y^{k}) & \\ =\frac{1}{n}\sum_{k=1}^{n}-E_{q_{\phi }(z^{k}|x^{k},y^{k})}[\text{log}\;p_{\theta }(x^{k}|z^{k},y^{k})] & \\ +\beta KL(q_{\phi }(z^{k}|x^{k},y^{k})\|p(z^{k}|y^{k}))+\alpha \|W{\|}_{1} & \end{array}$$

By training on all units at this stage, the model learns a general relationship between gene expression and cellular spatial coordinates. The GRN layers are optimized to capture an overall regulatory structure across the entire tissue. This trained model then serves as a foundation for Stage 2, where it is further fine-tuned by conditioning on a target location to infer more specific GRNs.

In Stage 2, we specialize the model to infer cell- or spot-specific GRNs. Since Stage 1 learns stable encoders and decoders, we freeze these components and update only the GRN layers. This stage is guided by two training strategies that leverage the relationship between spatial context and regulatory programs learned in stage 1. First, since stage 1 establishes a global mapping between gene expression patterns and spatial organization across the tissue, stage 2 conditions the model on a target spatial location to refine regulatory inference toward unit-specific GRNs. Specifically, to infer the GRN of a target unit *t*, we fix all spatial coordinates to $y^{t}$, forming $D_{2}^{t}={\left\{(x^{k},y^{t})\right\}}_{k=1}^{n}$. By conditioning on $y^{t}$, SVGRN models $p(x|y^{t})$ and updates the GRN toward a network specific to the target spatial context.

Second, we assume that nearby units share more similar GRNs than distant ones, particularly in developing tissues such as embryos. During development, GRNs vary smoothly across space due to coordinated patterning and gradual differentiation (Briggs *et al.* 2018, Belmonte-Mateos and Pujades 2021), which are driven by spatially distributed morphogen gradients that shape gene regulatory programs across the tissue (Bhatla and Sisodia 2023). Therefore, instead of relying solely on a single unit, Stage 2 leverages neighboring units to provide spatially weighted optimization signals for the target unit according to their spatial proximity, enabling robust estimation of microenvironment-specific regulatory programs. To implement this assumption, we introduce a kernel weight $K_{kt}$ into the loss function to quantify the contribution of a unit *k* when inferring the GRN of a target unit *t*, based on their spatial proximity. The kernel weight $K_{kt}$ is computed using a radial basis function kernel:


(10) $$\begin{array}{c} K_{kt}=\text{exp}\;\left(-\frac{{\left\|y^{k}-y^{t}\right\|}^{2}}{2\sigma^{2}}\right) \end{array}$$

where σ is a hyperparameter controlling the spatial range of neighbors contributing to the target GRN. Units closer to the target unit *t* are assigned higher loss weights, while distant units receive lower weights, emphasizing contributions from neighboring units. Combining these two strategies, the Stage 2 loss is defined as:


(11) $$\begin{array}{c} L_{\mathrm{stage}2}=\frac{1}{n}\sum_{k=1}^{n}K_{kt}L(x^{k},y^{t}) \end{array}$$

## 3 Results & discussion

### 3.1 Experiments on simulation datasets

Due to the lack of benchmark datasets with known spot/cell-specific GRNs, we evaluated SVGRN using simulated data generated by scMultiSim (Li *et al.* 2025a), which simulates scRNA-seq data with dynamic GRNs and spatially organized cells. To approximate diverse spatial transcriptomics settings, we varied key factors affecting GRN inference, including gene number and intrinsic noise. In scMultiSim, the intrinsic.noise parameter controls transcriptional noise, with higher values increasing the difficulty of GRN recovery (Li *et al.* 2025a).

To benchmark GRN inference performance, we adopted two evaluation metrics from the BEELINE framework: the early precision ratio (EPR) and the area under the precision–recall curve ratio (AUPRC ratio) (Pratapa *et al.* 2020). These metrics assess the accuracy of inferred networks by quantifying how well true regulatory edges are recovered. Early precision measures the fraction of true positives among the top *k* predicted edges, where *k* equals the number of edges in the ground-truth network. EPR is defined as the ratio of this early precision to that expected from random predictions, while the AUPRC ratio compares the inferred network’s AUPRC to a random baseline.

### 3.2 SVGRN captures dynamic GRNs through stage 2 training

**Figure 2:**
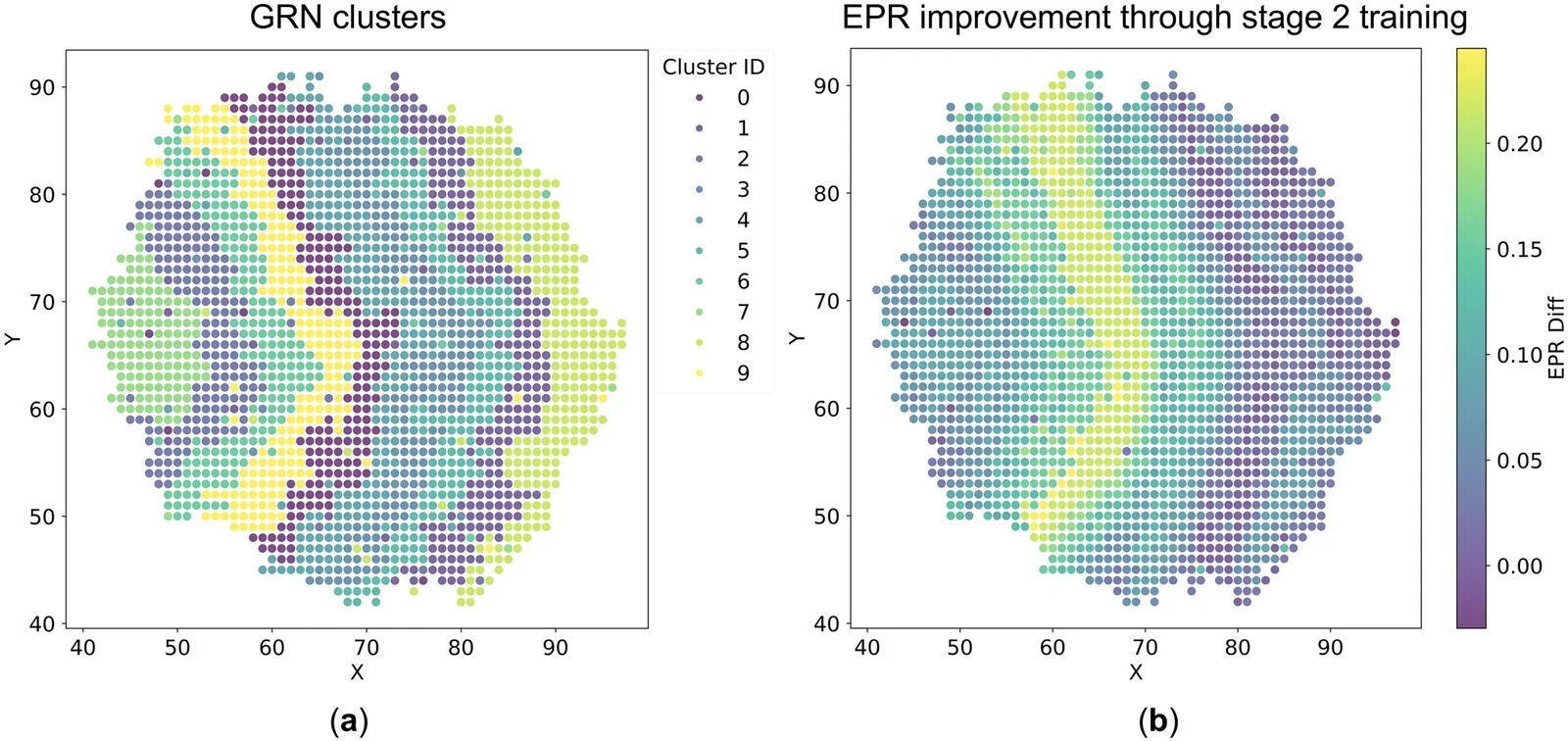
(a) The GRNs in all cells are clustered into 10 classes and the cluster ID for each cell is shown in their spatial layout. (b) For each cell, the difference between the initial EPR and that after the stage 2, spatial-unit-specific training is calculated and shown as a heatmap.

Figure 2 visualizes the spatial layout of a simulated dataset with 2000 cells, 110 genes, and intrinsic noise of 0.1. Starting from an initial GRN, we generated cell-specific GRNs by randomly adding or removing gene interactions, resulting in networks with slightly different edge compositions across cells. To enforce spatial organization, we used the “layers” parameter in scMultiSim, which places cells on a grid with higher probability near others with similar GRNs, forming layered clusters. As shown in Fig. 2a, each inferred cell-specific GRN was flattened into a feature vector and clustered using K-means. The resulting cluster labels were mapped back to the original spatial coordinates, revealing a layered spatial pattern consistent with the assumption that nearby cells tend to share similar regulatory networks.

Using scMultiSim ground-truth GRNs, we computed EPR for each cell before and after Stage 2. Figure 2b shows the spatial distribution of EPR improvements. Starting from a tissue-level GRN, SVGRN consistently refines cell-specific GRNs, with most cells showing improved EPR, demonstrating the effectiveness of the second-stage refinement. The improvement pattern aligns with the layered GRN structure, with similar gains within each layer, indicating that SVGRN captures spatially structured GRN variation through neighborhood information and enables effective fine-scale refinement of regulatory networks across space.

### 3.3 SVGRN outperforms benchmarking methods

**Figure 3:**
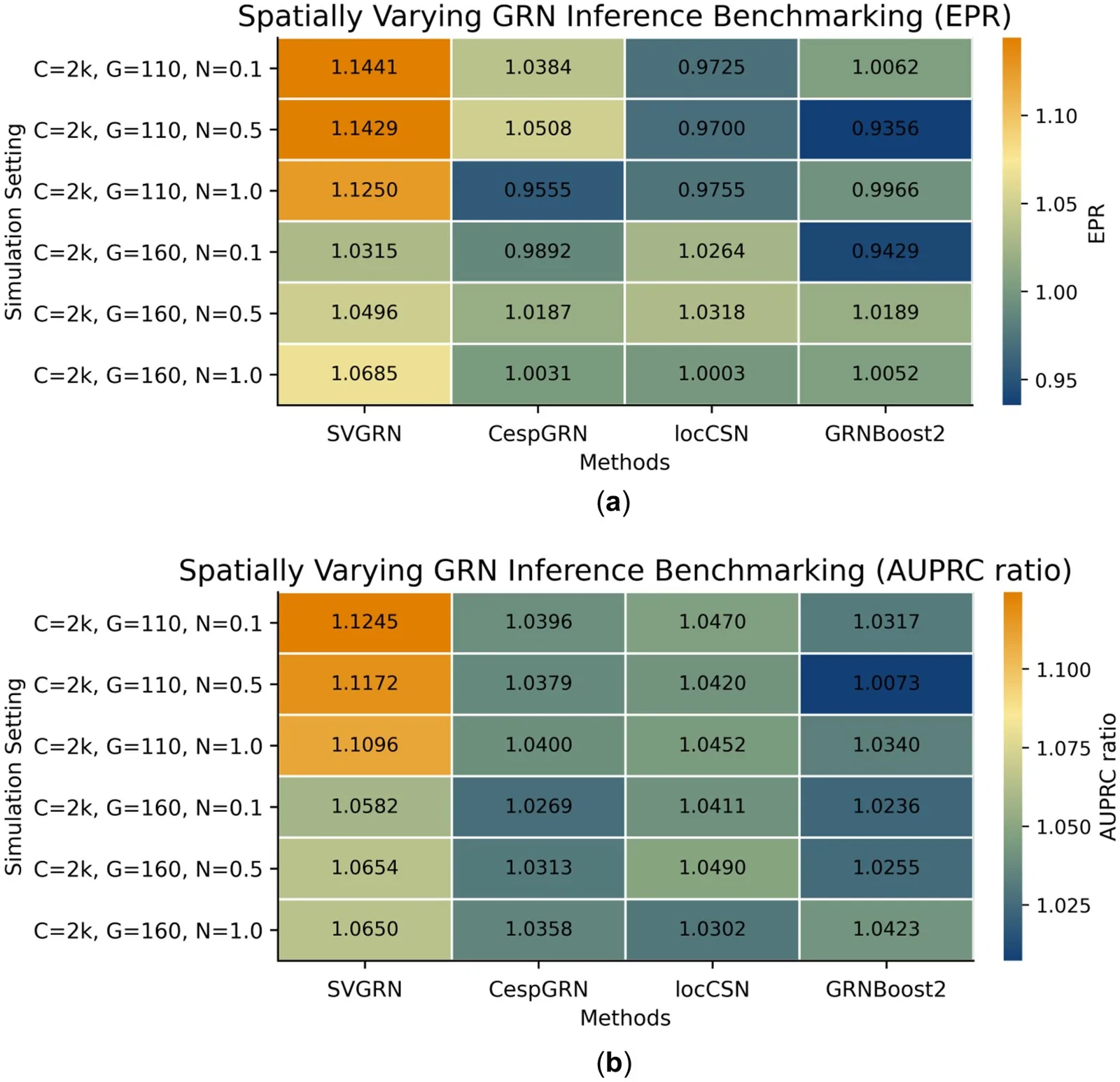
Benchmarking spatially varying GRN inference using EPR and AUPRC ratio across multiple simulated spatial transcriptomics settings.

We compared SVGRN with three representative state-of-the-art GRN inference methods based primarily on single-cell or spatially resolved gene expression, ensuring consistent input modalities without additional omics or dynamic information. TF–target pairs were provided as shared prior knowledge across all methods to ensure fair comparison. In addition, ground-truth GRNs for each cell were simulated with distinct regulatory edge structures to evaluate the ability to infer spatially varying GRNs. The baselines include CeSpGRN (Zhang *et al.* 2022) for spot- or cell-specific GRNs from spatial data, locCSN (Wang *et al.* 2021) for cell-specific GRNs, and GRNBoost2 (Moerman *et al.* 2019) for cell-type–level inference. As shown in Fig. 3, SVGRN consistently outperforms these methods in both EPR and AUPRC ratio across all simulation settings, demonstrating its superior ability to recover regulatory interactions.

Single-cell sequencing data are inherently noisy due to technical and biological variability (Kharchenko *et al.* 2014, Piwecka *et al.* 2023), and spatial transcriptomics introduces additional noise from experimental procedures (Wang *et al.* 2022). Such noise can obscure regulatory interactions and hinder GRN inference, making robustness essential. To evaluate this, we tested SVGRN on simulated datasets with noise levels of 0.1, 0.5, and 1.0. SVGRN maintains stable performance in both EPR and AUPRC ratio across all settings, demonstrating robustness to noise. We further evaluated datasets with larger gene sets, which introduce more potential regulatory edges and a more complex interaction landscape. As shown in Fig. 3, SVGRN consistently outperforms baseline methods for both 110 and 160 genes. These results highlight its robustness to both noise and increasing regulatory complexity. Additional ablation experiments, implementation details, and parameter sensitivity analyses are provided in Supplementary Notes 2, 4, and 6, available at supplementary material  *Bioinformatics Advances* online, respectively. Additional simulations evaluate the performance of SVGRN across datasets with larger numbers of cells and genes (Supplementary Note 7, available at supplementary material  *Bioinformatics Advances* online). Computational scalability and practical guidance for applying SVGRN to larger spatial transcriptomics datasets are discussed in Supplementary Note 8, available at supplementary material  *Bioinformatics Advances* online.

### 3.4 Experiments on the cell-based spatial transcriptomics dataset of mouse embryos

Unlike simulated data, true cell-specific GRNs are difficult to obtain experimentally due to biological complexity. Spatial transcriptomics enables the study of regulatory programs within their native spatial context. While spot-based methods such as Visium (Marx 2021) provide multi-cellular resolution, single-cell methods such as seqFISH (Lubeck *et al.* 2014) and MERFISH (Chen *et al.* 2015) offer finer characterization of cellular organization and neighborhood effects. Thus, cell-resolved spatial transcriptomics provides an ideal setting for inferring cell-specific GRNs and incorporating fine-scale spatial information. Here, we apply SVGRN to a seqFISH mouse embryo dataset (Lohoff *et al.* 2022) at the 8–12 somite stage to infer spatially varying GRNs. Early embryonic tissues exhibit gradual transitions in gene expression and regulatory programs, consistent with our assumption that nearby cells share similar GRNs.

**Figure 4:**
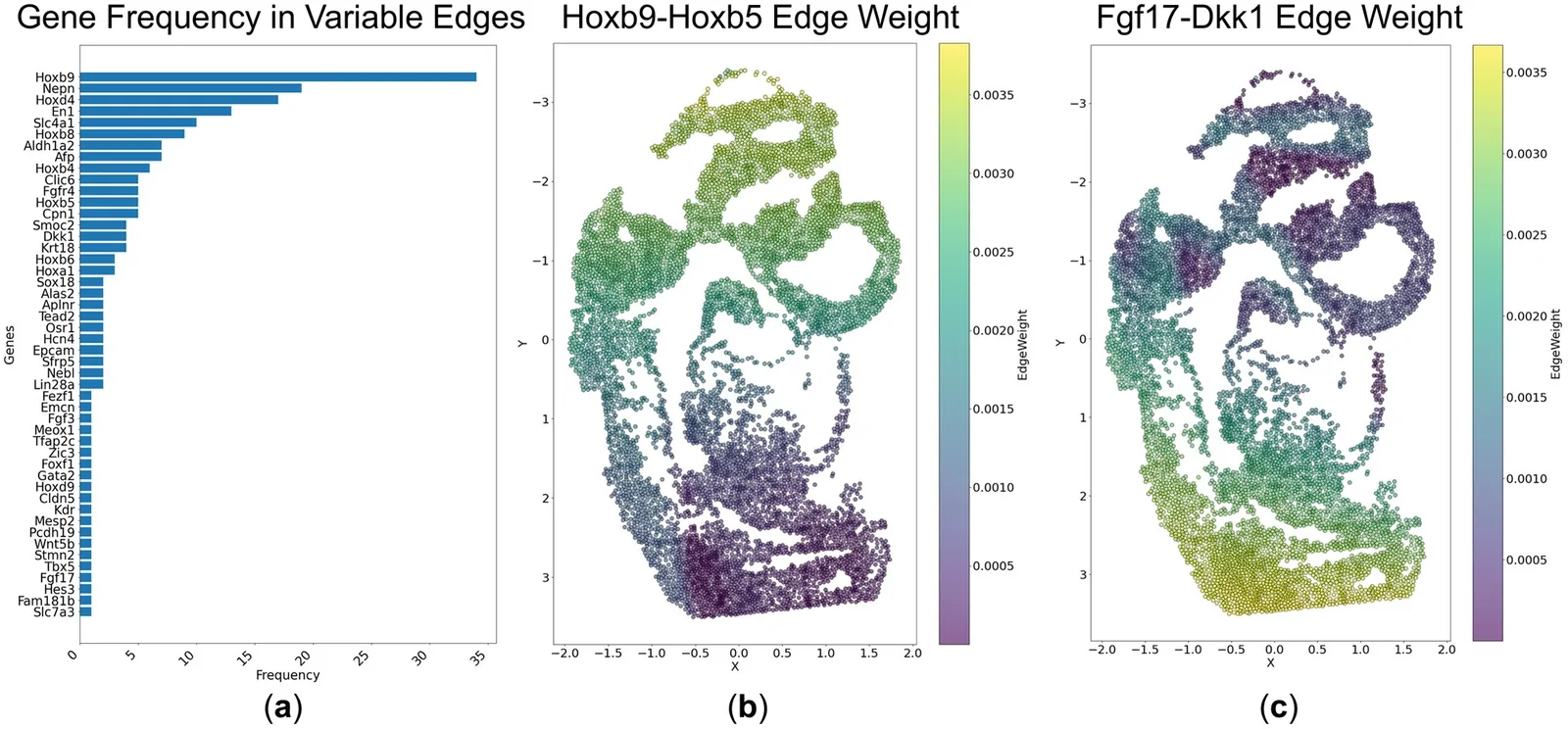
(a) The gene appearance frequency in the top 100 edges with the highest edge weight variance value across all the cells. (b) Hoxb9-Hoxb5 edge weight changing pattern in the mouse embryo tissue. (c) Fgf17-Dkk1 edge weight changing pattern in the mouse embryo tissue. In (b)-(c), the largest values of X correspond to the tail region, and the lowest values of Y correspond to the head region.

We trained SVGRN on a sagittal seqFISH section containing 6880 cells and 351 genes. From the inferred cell-specific GRNs, we computed the variance of each edge weight across cells to identify the most spatially variable interactions, which reflect regulatory relationships that change most across the tissue. The complete ranking of edge-weight variances is provided in Supplementary Data 1, available at supplementary material  *Bioinformatics Advances* online. Figure 4a shows the gene appearance frequencies among the top 100 most variable edges. Frequently identified genes, including Hoxb9, Hoxd4, En1, and Nepn, are known to exhibit region-specific functions during mouse embryonic development (Mallo *et al.* 2010, Altieri *et al.* 2015, Addeo *et al.* 2019, Hubert and Wellik 2023).

Analysis of representative interactions selected from the top 100 edges ranked by edge-weight variance shows that SVGRN captures spatially varying gene interactions associated with distinct body segments during early mouse embryonic development. Hox genes, including Hoxb9 and Hoxd4, are among the most spatially variable interactions. These genes regulate anterior–posterior patterning and coordinate vertebral, limb, and neural tube development (Mallo *et al.* 2010, Hubert and Wellik 2023), while also interacting within and across clusters to form regulatory domains critical for neural tube patterning and axial development (Forlani *et al.* 2003, Ahn *et al.* 2014). As shown in Fig. 4b, interaction weights among Hox genes exhibit clear spatial gradients, consistent with progressive neural tube differentiation from head to tail during the 8–12 somite stage. Higher interaction strengths in anterior regions reflect early neural tube formation, which extends posteriorly as development proceeds.

SVGRN also captures spatially structured interactions between Dkk1 and the Fgf8 subfamily. Dkk1 modulates Wnt–Fgf signaling and influences Fgf activity through both Wnt-dependent and independent mechanisms (Caneparo *et al.* 2007). These interactions are important for cell movement and limb bud formation, with Fgf8 activation in response to Dkk1 contributing to limb phenotypes (Mukhopadhyay *et al.* 2001). As shown in Fig. 4c, the Dkk1–Fgf17 interaction is stronger in posterior regions, consistent with hindlimb bud emergence.

### 3.5 Experiments on the spot-based spatial transcriptomics dataset of cSCC

Compared to cell-based data, spot-based spatial transcriptomics presents a more complex setting, as each spot may contain multiple cells. We applied SVGRN to a Visium dataset of human cutaneous squamous cell carcinoma (cSCC) (Ji *et al.* 2020), including tumor and adjacent normal regions. Although each spot aggregates multiple cells, spots are spatially constrained and often consist of nearby or related cell types or states, such that their expression profiles capture dominant regional regulatory programs shaped by local tissue context and neighboring spots. This allows SVGRN to leverage spatial coordinates and neighborhood information to learn informative regulatory patterns. Our results demonstrate its ability to infer spot-specific GRNs in mature tissues and capture gradual regulatory transitions from tumor core to leading edge and surrounding normal tissue, enabling analysis of spatially dynamic regulatory changes in the tumor microenvironment.

**Figure 5:**
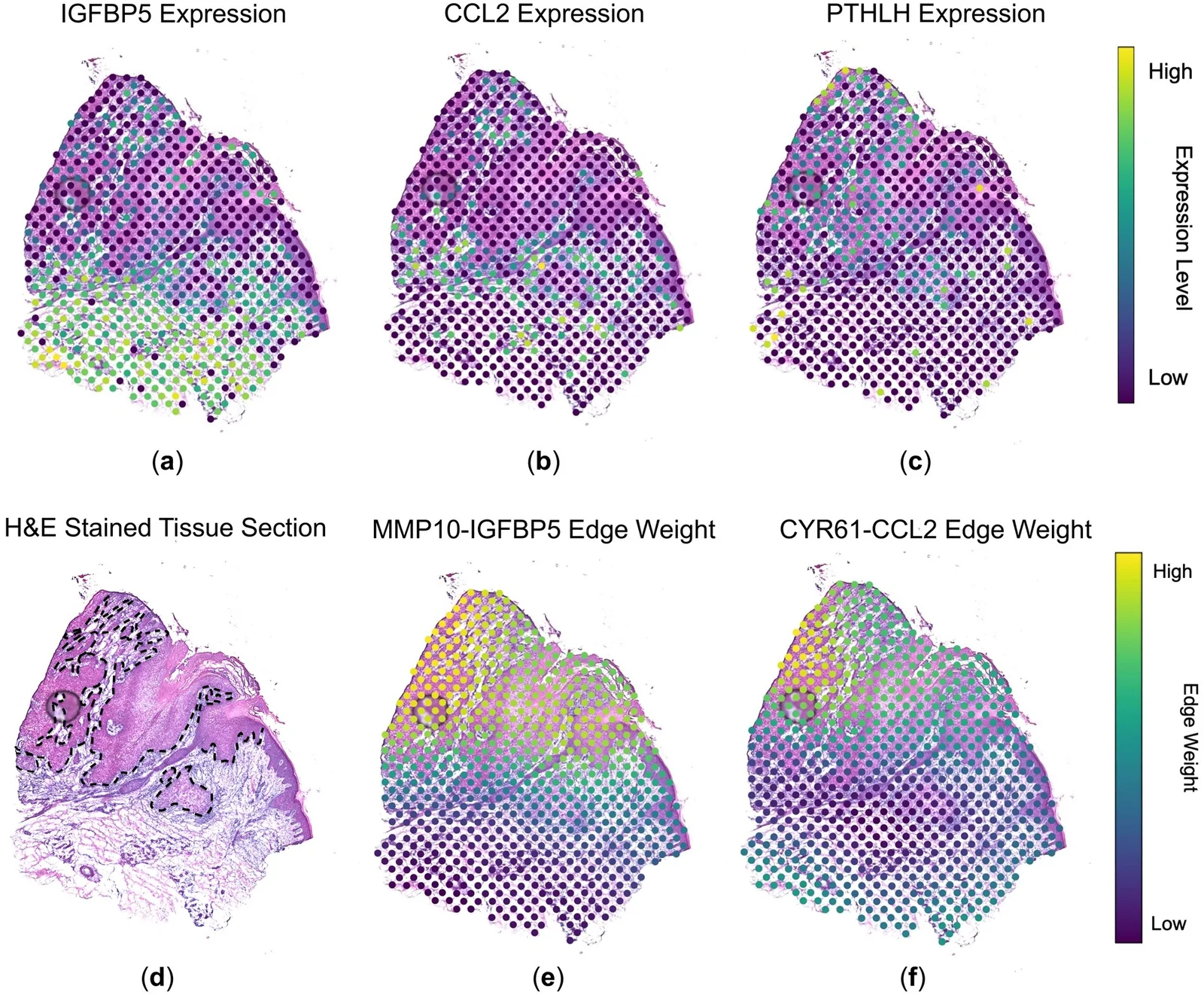
(a)–(c) Gene expression patterns of genes most frequently appeared in predicted high variable edges. (d) The H&E stained tissue section from the cSCC spatial transcriptomics dataset (Ji *et al.* 2020), with leading edge of tumor manually annotated (dotted lines). (e)–(f) Edge weight changing patterns of highly variable edges.

In this dataset, we analyzed a skin section containing 666 spatial spots on tissue and 17 139 genes. The corresponding H&E-stained tissue image is shown in Fig. 5d, indicating the spatial locations of tumor and adjacent normal regions. For model training, we focused on the top 40 marker genes from basal, cycling, differentiating, and tumor-specific keratinocyte (TSK) clusters shared between normal skin and cSCC.

From the top-ranked highly variable edges, SVGRN identifies genes with spatially distinct functions contributing to regulatory differences between tumor and normal regions. The complete ranking of edge-weight variances in the cSCC dataset is provided in Supplementary Data 2, available at supplementary material  *Bioinformatics Advances* online. Among the top 200 edges, IGFBP5, PTHLH, and CCL2 appear most frequently (42, 38, and 20 times). These genes show region-specific expression patterns (Fig. 5a–c) and are supported by prior studies. IGFBP5 is enriched in normal regions and suppresses tumor growth, while its downregulation promotes cancer progression (Waters *et al.* 2022, Lin *et al.* 2023). CCL2 is enriched near the tumor leading edge and may promote invasion and pre-metastatic niche formation (Jin *et al.* 2021). PTHLH is upregulated in cSCC and drives tumor progression via epithelial–mesenchymal interactions (Chang *et al.* 2017, Chen *et al.* 2021). Their spatial variation is reflected in their frequent involvement in highly variable edges, highlighting SVGRN’s ability to capture spatially dynamic regulation.

We further analyzed the spatial weight patterns of representative interactions selected from the top 200 most highly variable edges to examine how gene interaction strengths vary across space, providing insights into tumor-associated regulatory mechanisms. Matrix metalloproteinases (MMPs) are zinc-dependent endopeptidases that degrade extracellular matrix components and play key roles in tumor progression. They facilitate proteolytic cleavage of IGFBP5, thereby influencing the impact of IGFBP5 has in cancer (Waters *et al.* 2022). We examined the spatial distribution of the interaction between MMP10 and IGFBP5 across all spots. As shown in Fig. 5e, the interaction strength aligns with gene functions, with shifts corresponding to the spatial organization of tumor and normal regions.

Another notable interaction is between CCL2 and CYR61. As shown in Fig. 5f, the edge weight is elevated in tumor regions and decreases toward normal skin. CYR61 interacts with CCL2 to promote localized inflammation (Emre and Imhof 2014, Chen *et al.* 2017). In squamous cell carcinoma, CYR61 acts as a positive growth regulator (Kok *et al.* 2010), while CCL2 recruits immune cells that facilitate tumor progression (Yang *et al.* 2020, Kadomoto *et al.* 2021). CCL2 upregulation in tumors enhances immune cell recruitment, which in turn activates CYR61, forming a positive feedback loop that amplifies tumor-associated inflammation.

Analysis of predicted spot-specific GRNs demonstrates SVGRN’s ability to identify genes with spatially distinct functions and reveal location-dependent regulatory variation across tissue. Spatial edge-weight patterns highlight shifts in gene regulation underlying tumor progression and immune responses. These findings underscore the value of SVGRN for studying tumor development and microenvironment-driven cellular behavior. Additional analyses of biological datasets are provided in the Supplementary Material, available at supplementary material  *Bioinformatics Advances* online. Specifically, SVGRN was further applied to a Visium fallopian tube dataset to identify GRN-defined functional tissue niches (Supplementary Note 1 and Supplementary Data 3, available at supplementary material  *Bioinformatics Advances* online), and quantitative benchmarking using matched spatial transcriptomic and proteomic measurements further evaluated the biological relevance of the inferred GRNs against existing methods (Supplementary Note 5, available at supplementary material  *Bioinformatics Advances* online).

## 4 Conclusion

In this work, we propose SVGRN, a deep learning framework for inferring spatially resolved, unit-specific GRNs from spatial transcriptomics data. By jointly modeling gene expression and spatial coordinates, SVGRN captures regulatory interactions under two biologically motivated assumptions, enabling spot- or cell-specific inference while leveraging neighboring information. Experiments on simulated datasets demonstrate robustness to noise and increasing gene numbers. Applications to cell- and spot-based datasets further show its ability to capture spatially varying regulatory patterns across technologies and resolutions.

While SVGRN provides an effective framework for inference of spatially varying GRNs, several limitations should be noted. Although the proposed framework is motivated by a nonlinear SEM formulation, the current implementation is realized as a CVAE model. As a probabilistic generative model, the CVAE is designed to learn statistical dependencies between gene expression, spatial context, and latent regulatory representations rather than enforcing SEM-specific causal constraints. Consequently, the inferred GRNs should be interpreted as context-specific regulatory relationships instead of directed causal interactions. In addition, the current RBF-based neighborhood weighting assumes that nearby units tend to share similar regulatory programs. Near sharp tissue boundaries or highly heterogeneous interfaces, more flexible neighborhood definitions may further improve the modeling of abrupt spatial variation. Future work will extend the framework toward causal modeling of gene regulation, explore learnable or boundary-aware weighting schemes, and integrate additional signaling processes such as cell–cell communication.

## Supplementary Material

vbag230_Supplementary_Data

## Data Availability

The seqFISH mouse embryo dataset underlying this article is available at the Spatial Mouse Atlas 2020 repository at https://content.cruk.cam.ac.uk/jmlab/SpatialMouseAtlas2020/. The cSCC spatial transcriptomics dataset is available in the Gene Expression Omnibus (GEO) at https://www.ncbi.nlm.nih.gov/geo/, and can be accessed with accession number GSE144240; sample P2_ST_rep1 was used in this study. The fallopian tube dataset is available through the HuBMAP Program at https://hubmapconsortium.org, and the dataset used in this study is available at https://portal.hubmapconsortium.org/browse/dataset/fa7fbcd8ae9219225f5df25e8c5e994e.
