# Supplementary material for: Spatially varying gene regulation network inference from spatial transcriptomics
Source: Bioinform Adv. 2026 Aug 14;6(1):vbag230. doi: 10.1093/bioadv/vbag230 (PMC13615653; doi:10.1093/bioadv/vbag230)
Supplement: Supplementary file 1 [file vbag230_supplementary_data.zip › SVGRN_Supplementary_Material_Clean.pdf]

Supplementary Material

Supplementary Note 1: SVGRN helps identify and understand functional tissue niches through GRNs

We applied SVGRN to a Visium fallopian tube dataset from the HuBMAP Program (`https://hubmapconsortium.org`) to investigate spatially varying regulatory programs. Using predicted spot-specific GRNs, we characterized the cell identities and functional organization across the tissue, revealing regulatory differences beyond gene expression alone. The GRNs were clustered into five groups using k-means (Fig. S1(b)), uncovering distinct regulatory patterns corresponding to functional regions.

To characterize differential cellular activities, we extracted TF–target pairs from the TRRUST database [2] and computed the average interaction strength of each pair within and outside each cluster. This enabled identification of the most differentially regulated interactions. Differential TF-target interactions across the inferred GRN clusters are provided in Supplementary Data 3. As shown in Fig. S1(c), clusters 0, 2, 3, and 4 exhibit strongly differential interactions, whereas cluster 1 is primarily defined by weaker regulatory relationships. These differential TF–target patterns reveal cluster-specific regulatory programs and highlight functional tissue niches within the fallopian tube.

In cluster 0, the top interactions are enriched in cells near smooth muscle or fibroblast-like regions at the epithelial boundary. Key regulators such as KLF5, MYH11, and NR4A1–MMP2 are associated with epithelial and smooth muscle functions [3, 1, 15], while IGF2, ASH1L–TIMP1, and NCAM1 are linked to tissue regeneration, extracellular matrix remodeling, and cell–cell adhesion [6, 9]. In contrast, cluster 1 exhibits generally weaker regulatory interactions, suggesting a homeostatic epithelial state that prioritizes ciliogenesis and epithelial maintenance over inflammatory or remodeling programs.

Cluster 2 is enriched for inflammation and repair programs, with top interactions involving RELA, GADD45, and BCL2, consistent with inflammatory signaling, stress responses, and cell survival [19, 8, 11]. The presence of F8 further suggests a perivascular or endothelial-associated niche, considering its crucial role in the blood coagulation pathway [18]. Cluster 3 reflects regulatory programs associated with cellular stabilization and repair, including oxidative stress protection (GCLC), cell-cycle control (NR4A1, CDKN1B), and apoptosis modulation through DNMT1- TNFRSF10B [10, 5, 14, 20]. Cluster 4 represents a hypoxia-adapted perimuscular or epithelial interface. YY1-EPAS1 and HIF3A-EPAS1 indicate an active oxygen-sensing axis tuned to accommodate low-O_2 niches [13, 7].

Overall, these results demonstrate that SVGRN identifies spatially distinct groups characterized by unique regulatory programs that reflect their functional identities and tissue roles. This GRN-based perspective provides a more mechanistic view of tissue organization in the fallopian tube, not only through marker expression but also through the underlying regulatory logic that governs cellular activity.

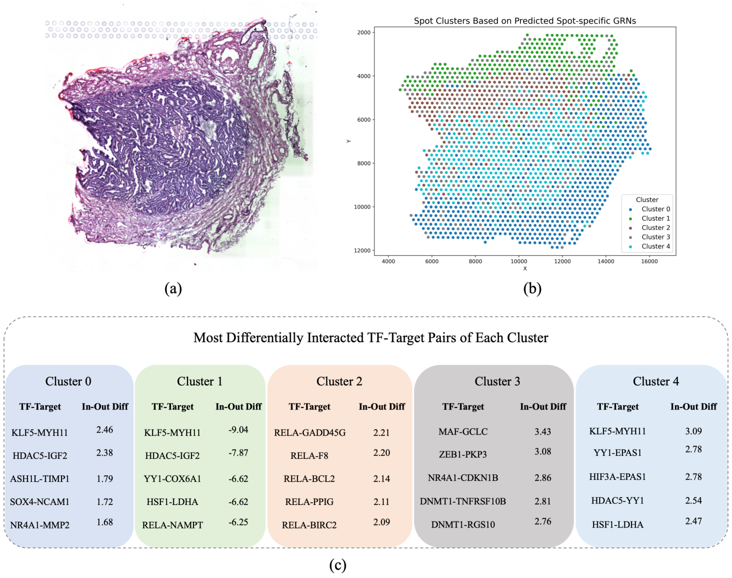

Figure S1: (a) The histology image of the fallopian tube sample. (b) Spots clustered by the predicted spot-specific GRNs. (c) The top differentially interacted TF-Target pairs in each cluster. *In-Out Diff* is the difference between the average interaction strength of spots in this cluster and out of this cluster, which are in 10^{-3} units.

Supplementary Note 2: Ablation experiments

To assess the impact of our two key strategies on cell- or spot-specific GRN learning, we conducted ablation experiments on two simulated datasets with noise levels of 0.1 and 0.5. In our model, we tuned the first stage GRN to more specific cell- or spot- level by fixing each cell's position to that of the target cell and by adding kernel weights to the loss function. In the ablations, we removed one or both of these components and compared the resulting average loss values, as shown in Table 1.

Without fixing the target position, spatial pattern modeling is removed, basically stopping SVGRN at the first stage that recovers a global GRN at the slide level, while removing kernel weights prevents the model from learning information from neighboring, more related cells. When both components are removed, the model fails to infer specific GRNs, resulting in identical networks across cells and the highest loss values in both datasets. As we incrementally reintroduce each assumption, we can observe significant loss reductions. This confirms that our assumptions not only allow for extending the whole-tissue GRN to single cells but also improve the reconstruction of gene expression based on spatial coordinates. By fixing coordinates alone, the model achieves a 0.13% and 6.6% loss reduction for noise levels 0.1 and 0.5, respectively. Adding kernel weights based on cell distances results in even greater reductions of 7.9% and 7.4% in the two cases.

Supplementary Note 3: Algorithm details

The pseudocode of the two-stage training of SVGRN is illustrated in Algorithm 1.

Algorithm 1 SVGRN Two-Stage Training for Cell-Specifc GRNs

Input: Gene expression $\{x^k\}_{k=1}^n$ and spatial coordinates $\{y^k\}_{k=1}^n$ for n cells, Hyperparameters α, β, σ, Epoch number E, Batch size B

Output: Cell-specific GRN matrixes $\{W^k\}_{k=1}^n$

▷ **Stage 1: Whole Tissue General GRN Training**

Randomly initialize all parameters including encoder parameters ϕ, decoder parameters θ, GRN matrix W

Define whole tissue dataset $\mathcal{D}_1 = \{(x^k, y^k)\}_{k=1}^n$

for each epoch $e = 1$ to E **do**

    for each data batch $b = 1$ to n/B **do**

        Give batch of gene expression $\{x^k\}$ and spatial coordinates $\{y^k\}$ to SVGRN model

        Calculate loss function L_{stage1}

        Update all model parameters ϕ, θ, W with gradients of L_{stage1}

    end for

end for

Save Stage 1 trained model

▷ **Stage 2: Cell-Specific GRN Training**

Load model from Stage 1 as initial parameters for Stage 2

for each target cell t **do**

    Freeze all model parameters θ, ϕ except for GRN matrix parameter W^t

    Define target-specific dataset $\mathcal{D}_2^t = \{(x^k, y^t)\}_{k=1}^n$ with fixed position y^t

    for each epoch $e = 1$ to E **do**

        for each batch $b = 1$ to n/B **do**

            Give batch of gene expression $\{x^k\}$ and fixed spatial coordinates $\{y^t\}$ to SVGRN model

            Calculate weighted loss function L_{stage2} using kernel weight K_{kt}

            Update GRN matrix parameter W^t with gradients of L_{stage2}

        end for

    end for

    Save the learned GRN matrix W^t for target cell t

end for

Table 1: Ablation experiment results showing the changes in loss values when removing the assumption implement components in the model.

**Dataset**			**Model**		
**Cell #**	**Gene #**	**Noise**	**Fix Target Pos (Assum. 1)**	**Add Kernel Weight (Assum. 2)**	**Loss Value**
					1.4846
2000	110	0.1	✓		1.4826
				✓	1.3673
			✓	✓	1.2652
					1.8651
2000	110	0.5	✓		1.7420
				✓	1.7259
			✓	✓	1.6961

Supplementary Note 4: Implementation details

The model was implemented in PyTorch. Parameters were optimized using the RMSprop optimizer with an initial learning rate of 1×10^{-4}. A step-based learning rate scheduler with a decay factor of 0.95 was applied to gradually reduce the learning rate during training. An L1 regularization term was added to the inferred adjacency matrix to encourage sparsity, implemented as a standard L1 penalty on edge weights. The model was trained with a batch size of 64.

To model spatial influence, we employed an RBF kernel under the assumption that nearby cells exhibit stronger interactions than distant cells. The bandwidth parameter was set to 4 for benchmarking experiments, 2 for cell-based experiments, and 5 for spot-based datasets. This parameter was adjusted across settings to reflect differences in spatial resolution and sampling density, ensuring that the kernel captures interactions at an appropriate spatial scale. Data preprocessing was performed prior to model training. Gene expression data were normalized using library size normalization followed by log transformation, and simulated datasets were preprocessed using the default scMultiSim pipeline.

Supplementary Note 5: Biological benchmarking using spatial transcriptomic and proteomic data

Since biologically validated cell-specific GRNs are unavailable for real tissues, it is difficult to directly evaluate the inferred GRNs or benchmark different GRN inference methods against ground-truth regulatory networks. From a biological perspective, TF activity is more closely associated with TF protein abundance than with TF mRNA expression alone, since TF proteins are the direct functional molecules regulating target gene expression. Therefore, if the inferred GRNs capture the underlying regulatory relationships, the TF activities estimated from the inferred GRNs should better agree with the independently measured TF protein abundance than the corresponding TF gene expression. Based on this rationale, we utilized the CosMx SMI Human Breast FFPE spatial multiomics dataset [4], which simultaneously measures transcriptomic and proteomic profiles from the same cells at subcellular resolution, to benchmark the ability of different GRN inference methods to recover biologically meaningful TF activities from transcriptomic data.

From the 64-plex proteomics panel, we selected the five TF proteins that also have corresponding genes in the transcriptomic data, including TP53 (p53), TCF7, RELA (NF-κB p65), CTNNB1 (β-catenin), and FOXP3. From the transcriptomic data, we selected three representative tissue regions, each defined by one or more fields of view (FOVs) and containing approximately 1,000–1,500 cells. For each region, approximately 50 highly variable genes that overlapped with the prior human TF–target pairs in TFLink [12] were selected for GRN inference. The RNA expression matrix was normalized by library size to 10,000 counts per cell, followed by log transformation.

Consistent with the simulation experiments, we benchmarked SVGRN against GRNBoost2, locCSN, and CeSpGRN. For all methods, only the human TF–target pairs from TFLink [12] supported by *small-scale evidence*, representing experimentally validated direct TF–target interactions, were used as prior knowledge. We then estimated the activity of each TF in every cell based on the inferred regulatory strengths and the corresponding target gene expression to quantitatively evaluate whether the inferred GRNs capture the underlying spatial variation of transcriptional regulation. Specifically, the TF activity was calculated as

$$\text{Activity}_i(\text{TF}) = \frac{1}{n} \sum_{t \in \mathcal{T}(\text{TF})} W(\text{TF}, t)\, Z(X_{it}),$$

where $W(\text{TF}, t)$ denotes the inferred regulatory weight from TF to target gene t, $\mathcal{T}(\text{TF})$ denotes the target genes of the TF, X_{it} denotes the expression of target gene t in cell i, and $Z(\cdot)$ represents the gene-wise z-score normalization across all cells.

To quantitatively evaluate the biological validity of the inferred cell-specific GRNs, we examined whether the inferred TF activities better reflected the independently measured TF protein expression than the corresponding TF gene expression. For the cell-specific GRN inference methods (SVGRN, locCSN, and CeSpGRN), we calculated the improvement in Spearman correlation between the inferred cell-specific TF activities and the measured TF protein intensity over the Spearman correlation between the TF mRNA expression and the corresponding TF protein intensity. This improvement quantifies whether the inferred GRNs can recover protein-level TF activities from transcriptomic data through the inferred regulatory mechanisms. For the cell-type GRN inference method, GRNBoost2, a direct cell-by-cell comparison is not applicable because all cells within the same cluster share an identical inferred GRN. To ensure a fair comparison while avoiding identical predicted TF activities across all cells, we first clustered cells into six groups within each selected region using K-means clustering and then applied GRNBoost2 to each cluster.

In SVGRN, the RBF kernel controls the neighborhood weighting of each target cell through the kernel bandwidth σ. Since the CosMx dataset contains cells with non-uniform spatial spacing, unlike the regular grid-like layouts in Visium and the simulated datasets, we further evaluated an adaptive-weight variant of SVGRN (SVGRN_adp) under this setting. In SVGRN_adp, the RBF kernel bandwidth σ is dynamically determined based on the distance to the target cell's kth nearest neighbor, whereas in the original SVGRN, σ is fixed and calculated from the mean distance to the kth nearest neighbor across all cells. In the biological benchmarking experiments, k was set to 5.

As shown in Fig. S2(a), both SVGRN and SVGRN_adp exhibited higher overall improvements over the RNA baseline across all evaluated TFs and tissue regions than the competing methods. In addition to achieving higher average improvements, the two SVGRN-based methods also showed predominantly positive improvement distributions, indicating that the inferred TF activities generally agreed better with the independently measured TF protein intensity than the corresponding TF gene expression alone. Averaging the improvements within each tissue region (Fig. S2(b)) further

demonstrated that SVGRN and SVGRN_adp consistently achieved the largest mean improvements across all three evaluated regions. Similarly, averaging the improvements across tissue regions for each individual TF (Fig. S2(c)) showed that SVGRN and SVGRN_adp consistently achieved the best performance for four of the five evaluated TFs. In contrast, all methods exhibited limited or negative improvements for CTNNB1. This may reflect the fact that CTNNB1 primarily functions as a transcriptional co-activator whose regulatory activity is strongly controlled by post-translational mechanisms [16, 17], making its activity more difficult to infer solely from transcriptomic regulatory relationships.

These results demonstrate that the GRNs inferred by SVGRN enable the estimated TF activities to achieve higher agreement with independently measured TF protein intensity than TF gene expression, indicating that the inferred regulatory relationships capture biologically meaningful information beyond the transcriptional abundance of individual TFs. Furthermore, the consistent improvements observed across multiple tissue regions and transcription factors suggest that incorporating spatial context enables SVGRN to recover biologically meaningful cell-specific regulatory programs that cannot be captured by gene expression alone.

Supplementary Note 6: Parameter sensitivity analysis

To evaluate the robustness of SVGRN with respect to hyperparameter selection, we conducted a sensitivity analysis on the simulated dataset consisting of 55 genes, 2,000 cells, and a noise level of 0.1. We investigated three representative hyperparameters that have the greatest influence on model performance, including the RBF kernel bandwidth (σ), the KL divergence weight (β), and the sparsity coefficient (α). For each hyperparameter, four representative values were evaluated. To isolate the effect of each hyperparameter, only one hyperparameter was varied during the Stage 2 training, which is responsible for inferring the cell-specific GRNs, while all remaining settings were fixed to their default values. The Stage 1 training was performed using the default hyperparameter settings in all experiments. The inferred GRNs were evaluated using both the EPR and the AUPRC ratio.

As shown in Fig. S3, SVGRN exhibits stable performance across a broad range of parameter values. Although moderate performance variations can be observed, the default parameter settings consistently achieve competitive or the best performance under both evaluation metrics. In particular, the model demonstrates limited sensitivity to the kernel bandwidth, indicating that the proposed neighborhood weighting strategy is robust to reasonable bandwidth choices. Similar trends are observed for both the KL divergence weight and the sparsity coefficient, suggesting that the proposed framework maintains stable performance across a reasonable range of hyperparameter settings for the loss function.

Supplementary Note 7: Simulation benchmarking across larger dataset scales

To further evaluate the robustness and scalability of SVGRN under different dataset scales, we conducted additional simulation benchmarking experiments on datasets with larger numbers of cells and genes. Specifically, we investigated two aspects separately. First, to evaluate the scalability with respect to cell number, we generated simulated datasets containing 110 genes with 3,000 and 4,000 cells, respectively. Second, to evaluate the robustness under increasing network complexity,

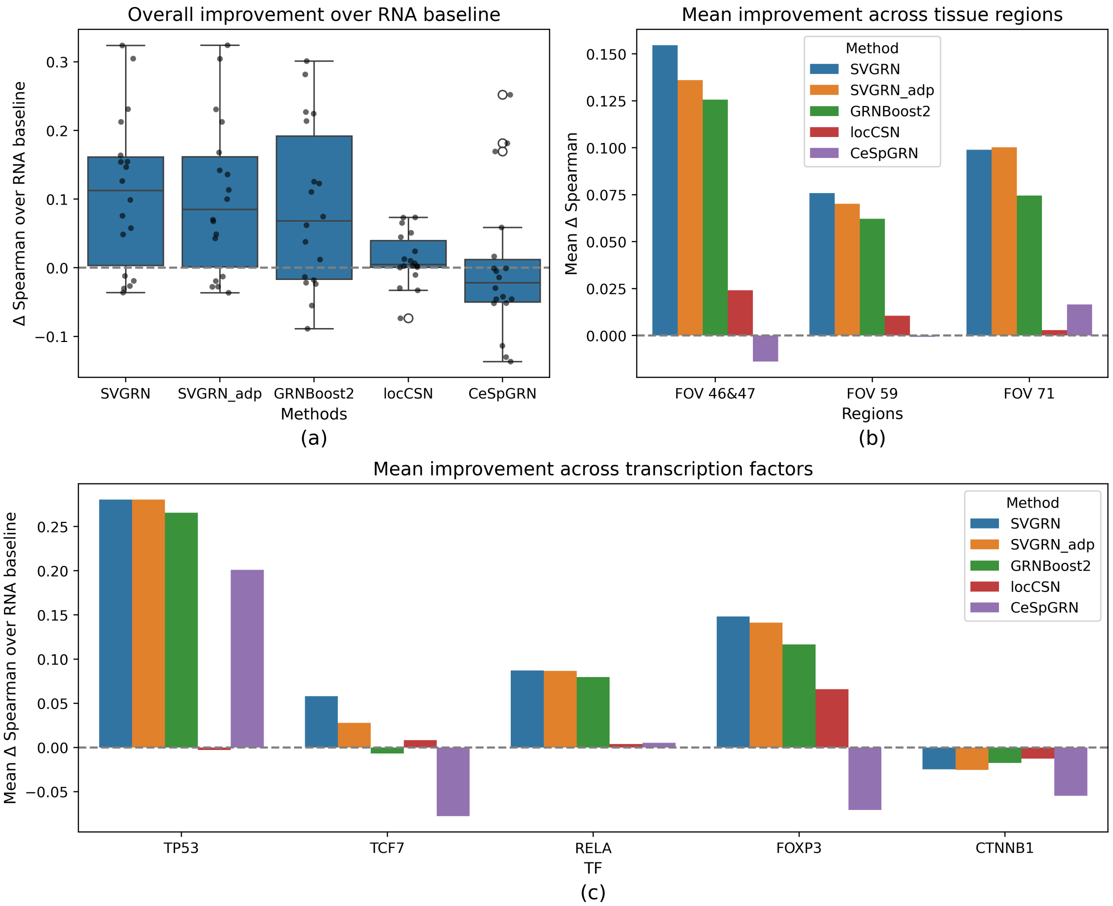

Figure S2: Biological benchmarking of GRN inference methods using the CosMx spatial transcriptomic-proteomic dataset. (a) Overall improvement in Spearman correlation between inferred TF activities and TF protein intensity over the RNA baseline. Each dot represents one TF in one tissue region (5 TFs $\times$ 3 regions, 15 samples in total). (b) Mean improvement across tissue regions. (c) Mean improvement across transcription factors. Positive values indicate that the inferred TF activities exhibit stronger agreement with independently measured TF protein intensity than the corresponding TF gene expression.

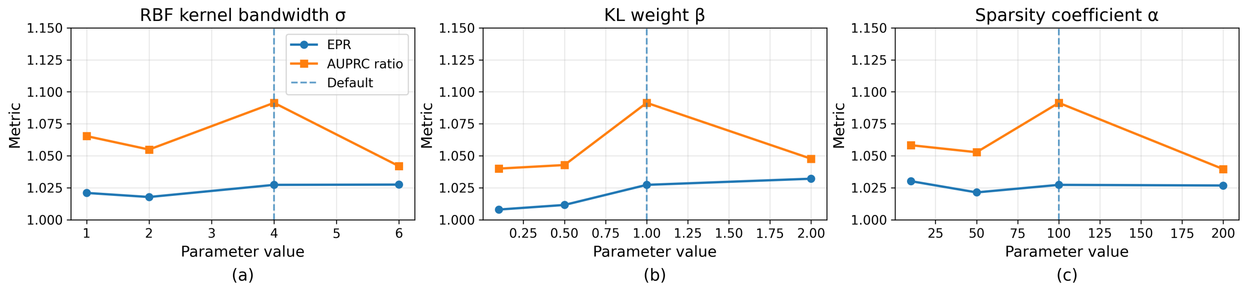

Figure S3: Sensitivity analysis of SVGRN with respect to the RBF kernel bandwidth (σ), KL divergence weight (β), and sparsity coefficient (α). The dashed vertical line indicates the default parameter setting used throughout this study. Performance was evaluated using the EPR and AUPRC ratio.

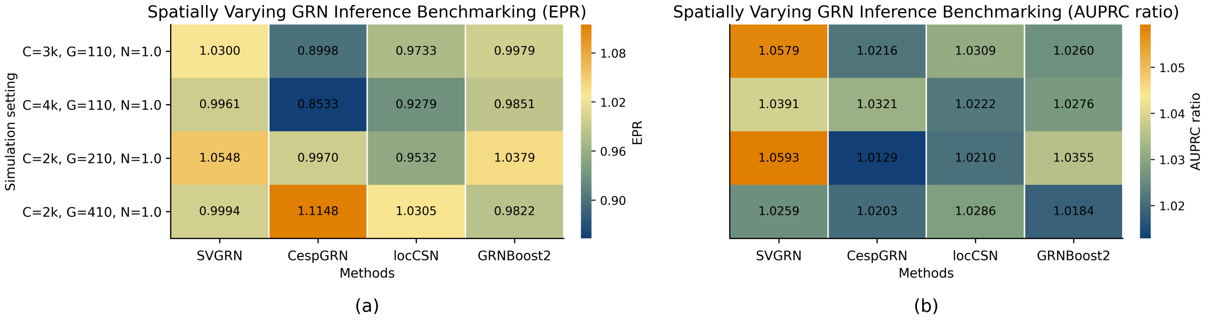

Figure S4: Performance comparison of SVGRN and existing GRN inference methods on additional simulated datasets with larger numbers of cells and genes. The additional datasets include simulations with increased cell numbers (3,000 and 4,000 cells, 110 genes) and increased gene counts (210 and 410 genes, 2,000 cells), all evaluated under a noise level of 1.0. (a) Early Precision Ratio (EPR). (b) AUPRC ratio. Across the evaluated dataset scales, SVGRN achieves competitive performance and obtains the best results in several settings.

we generated simulated datasets containing 210 and 410 genes while fixing the number of cells at 2,000. All additional experiments were conducted under the challenging noise level of 1.0, and all model hyperparameters were kept identical to those used in the main simulation experiments.

Consistent with the main benchmarking experiments, we compared SVGRN with GRNBoost2, CeSpGRN, and locCSN using both the EPR and AUPRC ratio. The complete benchmarking results are summarized in Fig. S4. As shown in Fig. S4, SVGRN consistently achieved the best overall performance on the datasets with increased cell numbers. For both the 3,000-cell and 4,000-cell datasets, SVGRN outperformed all competing methods in terms of both the EPR ratio and the AUPRC ratio, demonstrating that the proposed framework scales well with increasing numbers of cells.

Increasing the number of genes makes GRN inference substantially more challenging because the number of potential regulatory interactions grows rapidly with network size. Nevertheless, SVGRN maintained competitive performance on the larger gene sets. For the dataset containing 210 genes, SVGRN achieved the best performance among all methods on both evaluation metrics. For the most challenging dataset containing 410 genes, although the EPR ratio decreased compared with the smaller datasets, SVGRN still achieved a higher AUPRC ratio than GRNBoost2 and CeSpGRN while remaining competitive with locCSN. These results indicate that SVGRN maintains generally competitive performance across different dataset scales, including settings with increased gene numbers and inference complexity.

Supplementary Note 8: Scalability and practical guidance

All experiments were conducted on NVIDIA A40 GPUs. The computational cost of SVGRN mainly comes from Stage 2, where spatial-unit-specific GRNs are inferred independently for each target cell or spot. In contrast, Stage 1 learns the global GRN and is computationally efficient. In our experiments, Stage 1 finished within approximately 10 minutes for datasets containing up to 4,000 cells. Regarding the minimum training set size, we recommend using approximately 1,000 or more spatial units when analyzing around 50 genes to achieve relatively stable training, consistent with

the smallest dataset setting evaluated in our experiments.

Since Stage 2 is performed independently for each target unit, its runtime scales approximately linearly with the number of cells or spots and can be naturally parallelized by splitting target units into multiple subsets. In our implementation, we divided cells into four subsets and ran Stage 2 in parallel. In addition, the average runtime per target unit increased with the number of genes, providing a practical estimate of the computational cost of Stage 2 inference under different feature dimensions. The detailed runtime statistics are summarized in Fig. S5.

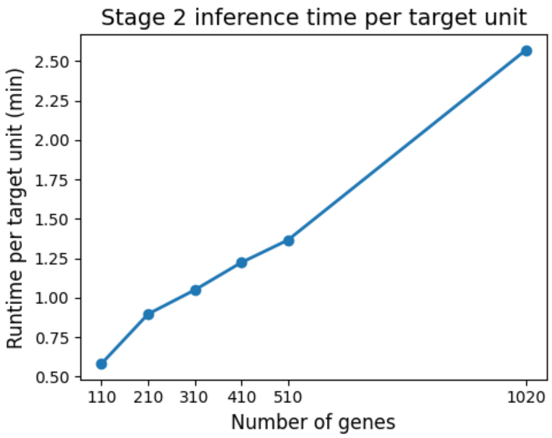

Figure S5: Average Stage 2 inference time per target unit under different gene numbers.

This design enables SVGRN to be efficiently scaled to larger spatial transcriptomics datasets through parallel execution of Stage 2 across target units. For large-scale spatial transcriptomics technologies such as MERFISH and Xenium, we recommend applying SVGRN in a region-wise or tile-wise manner when the dataset contains a very large number of cells. Because the inference for each target unit mainly depends on its local spatial neighborhood during Stage 2, spatial tiling with overlapping boundary regions can reduce computational burden while preserving local neighborhood information. Random subsampling of cells is not recommended when it disrupts local spatial neighborhoods.

Supplementary Data

The supplementary datasets accompanying this manuscript are listed below.

- **Supplementary Data 1.** Ranked edge-weight variances of inferred cell-specific GRNs in the mouse embryo dataset.
- **Supplementary Data 2.** Ranked edge-weight variances of inferred cell-specific GRNs in the cSCC dataset.
- **Supplementary Data 3.** Differential TF-target interactions across inferred GRN clusters in the Visium fallopian tube dataset.

References

[1] Kristie L Durst, Bart Lutterbach, Tanawan Kummalue, Alan D Friedman, and Scott W Hiebert. The inv (16) fusion protein associates with corepressors via a smooth muscle myosin heavy-chain domain. *Molecular and cellular biology*, 23(2):607–619, 2003.

[2] Heonjong Han, Jae-Won Cho, Sangyoung Lee, Ayoung Yun, Hyojin Kim, Dasom Bae, Sunmo Yang, Chan Yeong Kim, Muyoung Lee, Eunbeen Kim, et al. Trrust v2: an expanded reference database of human and mouse transcriptional regulatory interactions. *Nucleic acids research*, 46(D1):D380–D386, 2018.

[3] Shinichiro Hayashi, Ichiro Manabe, Yumi Suzuki, Frederic Relaix, and Yumiko Oishi. Klf5 regulates muscle differentiation by directly targeting muscle-specific genes in cooperation with myod in mice. *Elife*, 5:e17462, 2016.

[4] Shanshan He, Ruchir Bhatt, Carl Brown, Emily A Brown, Derek L Buhr, Kan Chantranuvatana, Patrick Danaher, Dwayne Dunaway, Ryan G Garrison, Gary Geiss, et al. High-plex imaging of rna and proteins at subcellular resolution in fixed tissue by spatial molecular imaging. *Nature biotechnology*, 40(12):1794–1806, 2022.

[5] Jacob A Herring, Weston S Elison, and Jeffery S Tessem. Function of nr4a orphan nuclear receptors in proliferation, apoptosis and fuel utilization across tissues. *Cells*, 8(11):1373, 2019.

[6] Che-Fang Hsu, Hsuan-Shun Huang, Pao-Chu Chen, Dah-Ching Ding, and Tang-Yuan Chu. Igf-axis confers transformation and regeneration of fallopian tube fimbria epithelium upon ovulation. *EBioMedicine*, 41:597–609, 2019.

[7] Xianping Huang, Weihe Zhou, and Yuefeng Zhang. Transcription factor yy1 enhances the stemness of lung cancer cells by stabilizing hypoxia factor hif-1α under a hypoxic microenvironment. *Environmental Toxicology*, 36(1):114–122, 2021.

[8] Arslon Humayun and Albert J Fornace Jr. Gadd45 in stress signaling, cell cycle control, and apoptosis. In *Gadd45 Stress Sensor Genes*, pages 1–22. Springer, 2022.

[9] Christina Kasper, Hanne Rasmussen, Jette S Kastrup, Shinji Ikemizu, E Yvonne Jones, Vladimir Berezin, Elisabeth Bock, and Ingrid K Larsen. Structural basis of cell–cell adhesion by ncam. *Nature structural biology*, 7(5):389–393, 2000.

[10] Cecile M Krejsa, Christopher C Franklin, Collin C White, Jeffrey A Ledbetter, Gary L Schieven, and Terrance J Kavanagh. Rapid activation of glutamate cysteine ligase following oxidative stress. *Journal of Biological Chemistry*, 285(21):16116–16124, 2010.

[11] Elisabeth Kucera, Franz König, Stefan Tangl, Karl Grosschmidt, Christian Kainz, and Gerhard Sliutz. Bcl-2 expression as a novel immunohistochemical marker for ruptured tubal ectopic pregnancy. *Human Reproduction*, 16(6):1286–1290, 2001.

[12] Orsolya Liska, Balázs Bohár, András Hidas, Tamás Korcsmáros, Balázs Papp, Dávid Fazekas, and Eszter Ari. Tflink: an integrated gateway to access transcription factor–target gene interactions for multiple species. *Database*, 2022:baac083, 2022.

[13] Esteban A Moya. Epas1/hif-2α a key regulator for chronic hypoxia across species. *The Journal of physiology*, pages 10–1113, 2022.

[14] Ilenia Pellarin, Alessandra Dall'Acqua, Andrea Favero, Ilenia Segatto, Valentina Rossi, Nicole Crestan, Javad Karimbayli, Barbara Belletti, and Gustavo Baldassarre. Cyclin-dependent protein kinases and cell cycle regulation in biology and disease. *Signal Transduction and Targeted Therapy*, 10(1):11, 2025.

[15] Ricardo Rodríguez-Calvo, Beatriz Ferrán, Judith Alonso, Ingrid Martí-Pàmies, Silvia Aguiló, Olivier Calvayrac, Cristina Rodríguez, and José Martínez-González. Nr4a receptors upregulate the antiproteinase alpha-2 macroglobulin (a2m) and modulate mmp-2 and mmp-9 in vascular smooth muscle cells. *Thrombosis and Haemostasis*, 113(06):1323–1334, 2015.

[16] Kinjal Shah and Julhash U Kazi. Phosphorylation-dependent regulation of wnt/beta-catenin signaling. *Frontiers in oncology*, 12:858782, 2022.

[17] Shuang Shang, Fang Hua, and Zhuo-Wei Hu. The regulation of β-catenin activity and function in cancer: therapeutic opportunities. *Oncotarget*, 8(20):33972, 2017.

[18] Arthur R Thompson. Structure and function of the factor viii gene and protein. In *Seminars in thrombosis and hemostasis*, volume 29, pages 011–022, 2003.

[19] Alicia A Tone, Carl Virtanen, Patricia Shaw, and Theodore J Brown. Prolonged postovulatory proinflammatory signaling in the fallopian tube epithelium may be mediated through a brca1/dab2 axis. *Clinical cancer research*, 18(16):4334–4344, 2012.

[20] Xiaofei Zhao, Xiangguo Liu, and Ling Su. Parthenolide induces apoptosis via tnfrsf10b and pmaip1 pathways in human lung cancer cells. *Journal of experimental & clinical cancer research*, 33(1):3, 2014.
